# Antimicrobial activity of aztreonam/avibactam and molecular characterization of Enterobacterales not susceptible to ceftazidime/avibactam and/or meropenem/vaborbactam from US medical centres (2016–2024)

**DOI:** 10.1093/jac/dkag308

**Published:** 2026-09-01

**Authors:** Helio S Sader, Krisztina M Papp-Wallace, John H Kimbrough, Dmitri Debabov, Rodrigo E Mendes, Mariana Castanheira

**Affiliations:** Element Iowa City (JMI Laboratories), North Liberty, IA, USA; Element Iowa City (JMI Laboratories), North Liberty, IA, USA; Element Iowa City (JMI Laboratories), North Liberty, IA, USA; AbbVie, North Chicago, IL, USA; Element Iowa City (JMI Laboratories), North Liberty, IA, USA; Element Iowa City (JMI Laboratories), North Liberty, IA, USA

## Abstract

**Background:**

Resistance to ceftazidime/avibactam and meropenem/vaborbactam has been increasing among carbapenem-resistant Enterobacterales (CRE) in US hospitals recently. We evaluated the activity of aztreonam/avibactam and comparators against isolates that were not susceptible to ceftazidime/avibactam and/or meropenem/vaborbactam and against MBL-producing Enterobacterales.

**Methods:**

A total of 80 927 Enterobacterales isolates were consecutively collected (one per patient) in 2016–2024 from 103 US medical centres and susceptibility tested by broth microdilution in a monitoring laboratory. Among those, 194 (0.24%) were not susceptible to ceftazidime/avibactam or meropenem/vaborbactam, 115 (0.14%) were not susceptible to both ceftazidime/avibactam and meropenem/vaborbactam, and 832 (1.0%) isolates were carbapenem resistant. These isolates were screened for carbapenemase (CBase) genes by WGS.

**Results:**

Aztreonam/avibactam was active against 91.9% of ceftazidime/avibactam–non-susceptible isolates (*n* = 160), 94.0% of meropenem/vaborbactam–non-susceptible isolates (*n* = 149), 98.1% of CRE and 98.4% of MBL producers (*n* = 125). Cefiderocol retained activity against 78.8% of ceftazidime/avibactam–non-susceptible isolates, 83.2% of meropenem/vaborbactam–non-susceptible isolates, 94.7% of CRE and 82.1% of MBL producers. A CBase was identified in 159 (82.0%) of isolates not susceptible to ceftazidime/avibactam or meropenem/vaborbactam and in 683 (80.1%) of CREs. NDM (*n* = 114; 58.8% of isolates) was the most common CBase type among isolates not susceptible to ceftazidime/avibactam or meropenem/vaborbactam. Notably, an MBL was identified in 36.1% (73/202) of CRE collected in 2023**–**2024.

**Conclusions:**

Aztreonam/avibactam demonstrated potent activity against isolates not susceptible to ceftazidime/avibactam and/or meropenem/vaborbactam as well as against CRE isolates, including MBL producers. The activities of other β-lactamase inhibitor combinations and cefiderocol were adversely affected by the alarming increase of MBL producers in some US hospitals.

## Introduction

The clinical approval of β-lactamase inhibitor combination (BLIC) agents that are stable to hydrolysis by carbapenemases, such as ceftazidime/avibactam, meropenem/vaborbactam and imipenem/relebactam, represented a significant advance in the treatment of infections caused by carbapenem-resistant Enterobacterales (CRE). However, these agents have some limitations on their spectrum of activity. For instance, meropenem/vaborbactam and imipenem/relebactam have reduced activity against OXA-48–like producers, and none of these three BLIC agents are active against MBL producers.^1^ Consequently, the activities of these BLICs are compromised in geographical regions and/or medical centres where CRE isolates that produce these enzymes, mainly MBLs, are prevalent. Moreover, the occurrence of MBL producers, especially New Delhi metallo-β-lactamase (NDM) producers, appears to be rapidly increasing in some US medical centres.^2–4^

The siderophore cephalosporin, cefiderocol, has recently been approved for clinical use and represents another valuable option for the treatment of CRE infections.^5^ Cefiderocol has improved stability against carbapenemases, including MBLs. Moreover, cefiderocol overcomes resistance mediated by porin loss or efflux pumps by being actively transported across the outer membrane by the iron transportation system of Gram-negative bacteria.^6^ However, a variety of mechanisms, generally acting in combination, have been reported to confer resistance to cefiderocol, including some β-lactamases, mutations affecting siderophore receptors, porin alterations, overexpression of efflux pumps, and PBP-3 modifications. Expression of multiple β-lactamases (especially NDM, OXA-427, some PER- and SHV-types of ESBL, and some KPC and AmpC variants that confer resistance to ceftazidime/avibactam) in combination with decreased permeability, appears to be the principal mechanism of acquired resistance to cefiderocol.^7^ Furthermore, data are scarce on cefiderocol activity against CRE isolates not susceptible to ceftazidime/avibactam or meropenem/vaborbactam.

Aztreonam/avibactam was initially approved in the European Union in April 2024 (https://www.ema.europa.eu/en/news/new-antibiotic-fight-infections-caused-multidrug-resistant-bacteria) and in the USA in February 2025 (https://www.accessdata.fda.gov/drugsatfda_docs/label/2025/217906Orig1s000lbl.pdf). This agent combines the stability against hydrolysis by MBLs conferred by aztreonam with the protection against serine β-lactamases provided by avibactam and has demonstrated to be highly active against CRE, including MBL producers.^8,9^ In this investigation we evaluated the activity of aztreonam/avibactam and comparator agents, including cefiderocol and imipenem/relebactam, against isolates not susceptible to ceftazidime/avibactam and/or meropenem/vaborbactam and against MBL producers from US medical centres.

## Methods

The bacterial isolates were collected via the International Network for Optimal Resistance Monitoring (INFORM) programme.^4^ A total of 80 927 Enterobacterales isolates were consecutively collected in 2016–2024 from 103 US medical centres. Only one isolate per infection episode was included. Among these isolates, 160 (0.20%) were not susceptible to ceftazidime/avibactam, 149 (0.18%) were not susceptible to meropenem/vaborbactam, 115 (0.14%) were not susceptible to both ceftazidime/avibactam and meropenem/vaborbactam, 194 (0.24%) were not susceptible to either ceftazidime/avibactam or meropenem/vaborbactam, and 832 (1.03%) isolates were CRE. Only bacterial isolates determined to be significant by local criteria as the reported probable cause of infection were included in the study. Species identification was confirmed by standard biochemical tests and/or using the MALDI Biotyper (Bruker Daltonics, Billerica, MA, USA).

All isolates were susceptibility tested in a monitoring laboratory [Element Iowa City (JMI Laboratories)] by the broth microdilution method as described by CLSI.^10^ Cefiderocol was tested in iron-depleted media.^10^ CLSI and/or US FDA breakpoint criteria were used to interpret MIC values unless noted.^11,12^ CRE were defined as displaying imipenem or meropenem MIC values of ≥4 mg/L and imipenem was not applied to *Proteus mirabilis* or indole-positive Proteeae due to their intrinsically elevated imipenem MIC values. All CRE isolates were screened for β-lactamase–encoding genes by applying genome sequencing and *in silico* screening, as previously described.^13^ The generated FASTQ files were assembled using SPAdes Assembler and subjected to proprietary software [Element Iowa City (JMI Laboratories)] for screening of β-lactamase genes.^14^ All carbapenemase genes included in the National Database of Antibiotic Resistant Organisms (https://www.ncbi.nlm.nih.gov/pathogens/antimicrobial-resistance/) were evaluated. Statistical analysis was performed using Epi Info^TM^ 7.2 (CDC, Atlanta, GA, USA).

## Results

Aztreonam/avibactam was active (MIC ≤4 mg/L) against 91.9% (MIC_50/90_, 0.25/4 mg/L) of ceftazidime/avibactam–non-susceptible isolates, 94.0% (MIC_50/90_, 0.25/4 mg/L) of meropenem/vaborbactam–non-susceptible isolates, 98.1% (MIC_50/90_, 0.25/1 mg/L) of CRE and 98.4% (MIC_50/90_, 0.12/1 mg/L) of MBL-producing isolates [Table 1 and Figure S1 (available as Supplementary data at *JAC* Online)]. Cefiderocol retained activity against 78.8% (MIC_50/90_, 2/16 mg/L) of ceftazidime/avibactam–non-susceptible isolates, 83.2% (MIC_50/90_, 2/16 mg/L) of meropenem/vaborbactam–non-susceptible isolates, 94.7% (MIC_50/90_, 1/4 mg/L) of CRE and 82.1% (MIC_50/90_, 2/16 mg/L) of MBL-producing isolates (Table 1). Moreover, aztreonam/avibactam was active against 92.1% of cefiderocol–non-susceptible isolates (*n* = 38), and cefiderocol was active against 81.2% of aztreonam/avibactam–non-susceptible isolates (*n* = 16).

**Table 1.** Antimicrobial susceptibility of resistant subsets

Antimicrobial	% Susceptible per CLSI and/or US FDA (no. of isolates)				
agent	CAZ/AVI-NS (160)	MEM/VAB-NS (149)	CAZ/AVI-NS and MEM/VAB-NS (115)	CRE (832)	MBL producers (125)
Aztreonam/avibactam	91.9	94.0	93.9	98.1	98.4
Ceftazidime/avibactam	0.0	22.8	0.0	84.3	5.6
Meropenem/vaborbactam	25.8	0.0	0.0	79.1	9.8
Imipenem/relebactam	13.5	6.7	2.6	74.8	2.4
Cefiderocol	78.8	83.2	80.9	94.7	82.1
Levofloxacin	24.4	18.8	20.9	25.8	19.2
Gentamicin	60.0	54.4	58.3	55.6	58.4
Amikacin	63.1	56.4	58.3	61.1	57.6

CAZ/AVI, ceftazidime/avibactam; CRE, carbapenem-resistant Enterobacterales; MEM/VAB, meropenem/vaborbactam; NS, non-susceptible.

Among non–β-lactam agents, amikacin was the most active compound against ceftazidime/avibactam–non-susceptible isolates (MIC_50/90_, 4/32 mg/L; 63.1% susceptible), meropenem/vaborbactam–non-susceptible isolates (MIC_50/90_, 4/>32 mg/L; 56.4% susceptible) and CRE (MIC_50/90_, 4/32 mg/L; 61.1% susceptible); whereas gentamicin (MIC_50/90_, 1/>8 mg/L; 58.4% susceptible) was slightly more active than amikacin (MIC_50/90_, 4/>32 mg/L; 57.6% susceptible) against MBL producers based on current CLSI breakpoints (Table 1).

The activities of aztreonam/avibactam and cefiderocol against isolates not susceptible to ceftazidime/avibactam and meropenem/vaborbactam (*n* = 115) varied among species (Table S1). Aztreonam/avibactam was highly active against *Klebsiella pneumoniae* (*n* = 49; 98.0% susceptible) and *Enterobacter cloacae* complex (*n* = 23; 100.0% susceptible), but showed lower activity against *Escherichia coli* (*n* = 24; 87.5% susceptible). Cefiderocol was active against 87.8% of *K. pneumoniae*, 91.7% of *E. coli*, and only 52.2% of *E. cloacae* species complex isolates not susceptible to both ceftazidime/avibactam and meropenem/vaborbactam (Table S1).

**Figure 1.**
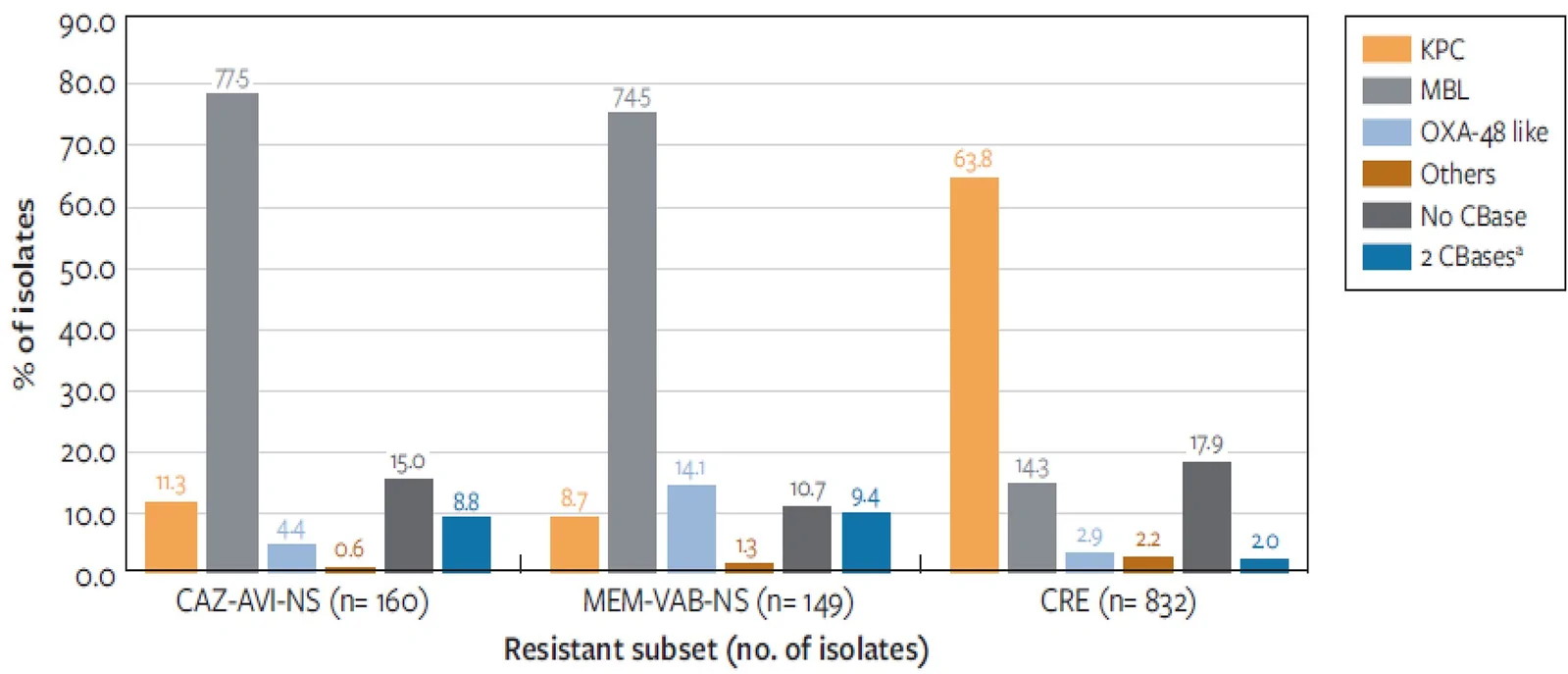
Distribution of carbapenemase types. ^a^Two carbapenemase genes were identified in 17 isolates, including NDM plus KPC (7), NDM plus OXA-48–like (7), KPC-2 plus GES-6 carbapenemase (1), KPC-3 plus IMP-4 (1), and KPC-6 plus KPC-10 (1). CAZ-AVI, ceftazidime/avibactam; CRE, carbapenem-resistant Enterobacterales; MEM-VAB, meropenem/vaborbactam; NS, non-susceptible.

**Figure 2.**
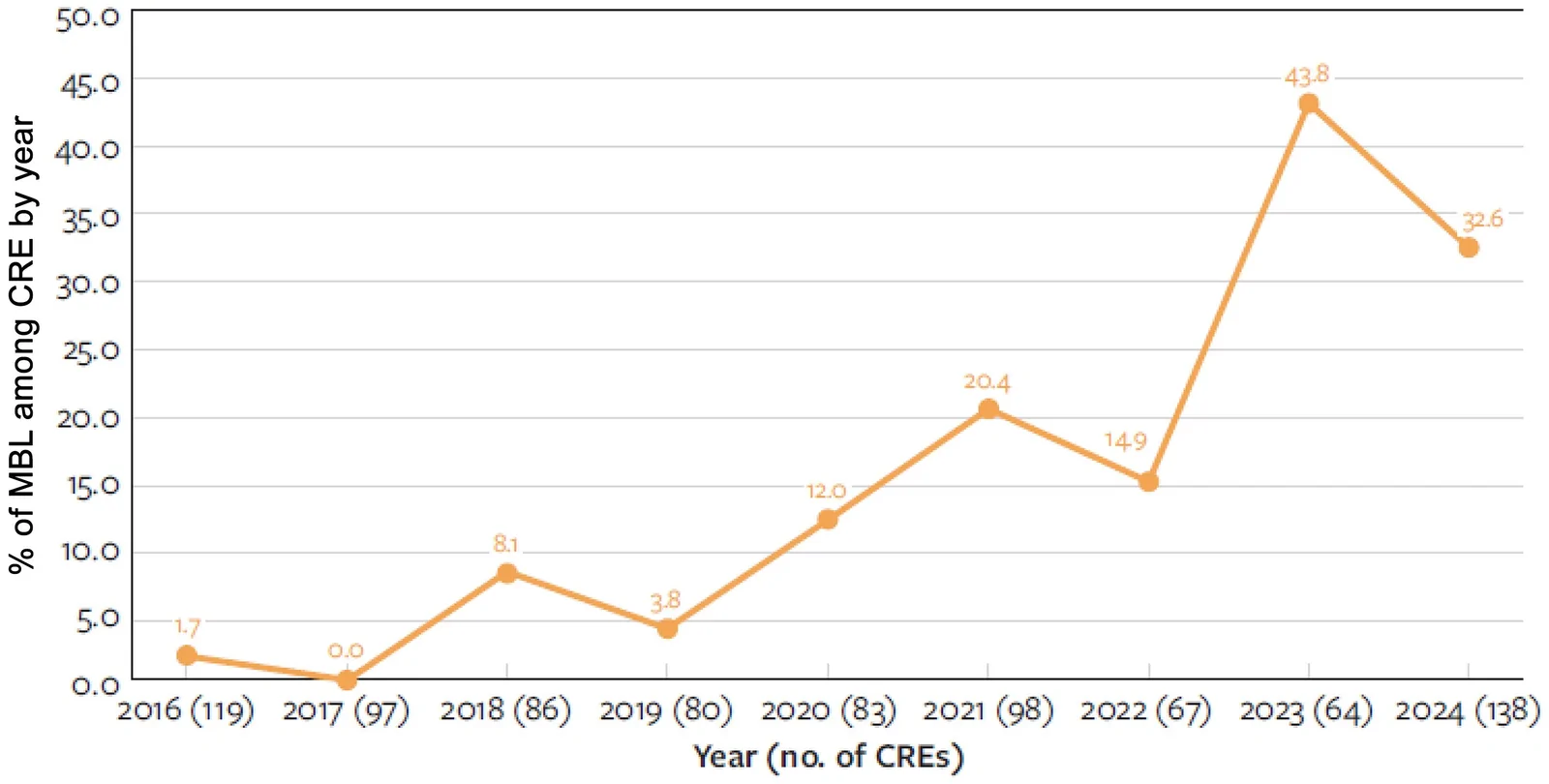
Frequency of MBL producers among CRE over the years.

A carbapenemase (CBase) was identified in 136 (85.0%) isolates not susceptible to ceftazidime/avibactam, in 133 (90.6%) isolates not susceptible to meropenem/vaborbactam, and in 683 (80.1%) CREs. NDM was the most common CBase type among isolates not susceptible to ceftazidime/avibactam (*n* = 112; 70.0%) and isolates not susceptible to meropenem/vaborbactam (*n* = 106; 71.1%), whereas KPC (*n* = 531; 63.8% of CREs) was the most common CBase type among CRE (Figure 1). NDM was identified in 113 (13.6%) CRE (Figure 1). An MBL was identified in 77.5% of isolates not susceptible to ceftazidime/avibactam and in 74.5% of isolates not susceptible to meropenem/vaborbactam (Figure 1). Notably, the frequency of MBL producers among CRE increased significantly from 1.7% in 2016 to 43.8% in 2023 and decreased to 32.6% in 2024 (Figure 2). Moreover, the Cochran–Armitage trend test indicated that the prevalence of MBL producers among CRE isolates trended markedly upwards during the study period (*P* < 0.001).

Aztreonam/avibactam was active against 92.1% of cefiderocol–non-susceptible isolates (*n* = 38), and cefiderocol was active against 81.2% of aztreonam/avibactam–non-susceptible isolates (*n* = 16). Among aztreonam/avibactam–non-susceptible CRE (*n* = 16), a carbapenemase was identified in five isolates (31.3%): one *E. cloacae* species complex with NMC-A, two *E. coli* with NDM-5, and two *Serratia marcescens* isolates, one with KPC-3 and one with SME-4. In contrast, 27 of 38 (71.1%) cefiderocol–non-susceptible isolates produced a carbapenemase, including NDM-1 or NDM-5 (19 isolates), KPC-3 or KPC-4 (4), KPC-65 plus NDM-5 (1), NDM-1 plus OXA-232 (1), IMI-4 (1) and VIM-1 (1).

## Discussion

The introduction of new antimicrobial agents in clinical practice is generally followed by the emergence of resistance, especially among Gram-negative bacteria.^15^ Thus, comprehensive surveillance programmes are pivotal for detecting the emergence of resistance and providing susceptibility data on organisms resistant to the new agent.^16^ The INFORM programme started monitoring the *in vitro* activities of ceftazidime/avibactam and meropenem/vaborbactam against Enterobacterales from US medical centres years before these drugs were approved for clinical use by the US FDA.^17,18^ Although these two BLICs exhibited almost complete activity against Enterobacterales for several years after their clinical approval and are still very active against CRE in the vast majority of US medical centres, resistance recently has started increasing in some hospitals.^3,4^ Resistance to imipenem/relebactam and cefiderocol has also been increasingly reported.^19,20^ Thus, it became important to generate susceptibility data on large collections of clinical isolates that have acquired resistance to these newer agents.

In the present investigation we evaluated the antimicrobial susceptibility of >80 000 Enterobacterales that were consecutively collected from 103 US medical centres. We detected only 194 (0.24%) isolates that were not susceptible to either ceftazidime/avibactam or meropenem/vaborbactam, and the most active agents against these isolates were aztreonam/avibactam (92.3% susceptible) and cefiderocol (81.3% susceptible). We could not find published data on the antimicrobial susceptibility of Enterobacterales not susceptible to ceftazidime/avibactam or meropenem/vaborbactam from US medical centres for comparison, but our results corroborate data from other geographical regions. Lee *et al*. reported aztreonam/avibactam activity against 3824 ceftazidime/avibactam-resistant CRE isolates collected from 307 medical centres worldwide, mainly from Asia, Europe and Latin America in 2019–2023.^2^ Only 12 isolates in the collection were from North America. Aztreonam/avibactam was very active (>96% susceptibility) against most Enterobacterales species. CRE species with lower aztreonam/avibactam susceptibility were *E. coli* (78.1% susceptible), *P. mirabilis* (86.0% susceptible) and *Providencia stuartii* (89.5% susceptible), and most aztreonam/avibactam–non-susceptible isolates were from India and China.^2^ Similarly, among the collection of isolates not susceptible to ceftazidime/avibactam and meropenem/vaborbactam evaluated in this investigation, susceptibility to aztreonam/avibactam was lower in *E. coli* (87.5% susceptible) compared with *K. pneumoniae* (98.0% susceptible) and *E. cloacae* species complex (100.0% susceptible; Table S1).

This study has some limitations. Not all medical centres participated in the INFORM programme each year. Medical centres that withdraw from the programme during the investigation were generally replaced with medical centres with similar characteristics from the same geographical region; however, antimicrobial susceptibility rates and epidemiology could certainly vary between two such centres. Despite these limitations, the results presented here provide valuable new information on the epidemiology of an important subset of MDR Enterobacterales.

In summary, the results of this investigation clearly showed that aztreonam/avibactam retains activity against Enterobacterales not susceptible to ceftazidime/avibactam and/or meropenem/vaborbactam from US medical centres. Our results also showed that the frequency of MBL producers among CRE isolates increased markedly during the study period (2016–2024). Aztreonam/avibactam remains very active against CRE isolates, including MBL producers; however, the activities of ceftazidime/avibactam, meropenem/vaborbactam and imipenem/relebactam have been adversely affected by the growing incidence of MBL producers in US medical centres.

## Supplementary Material

dkag308_Supplementary_Data

## Supplementary data

Figure S1 and Table S1 are available as Supplementary data at *JAC* Online.
