# Supplementary material for: Antimicrobial activity of aztreonam/avibactam and molecular characterization of Enterobacterales not susceptible to ceftazidime/avibactam and/or meropenem/vaborbactam from US medical centres (2016–2024)
Source: J Antimicrob Chemother. 2026 Sep 1;81(10):dkag308. doi: 10.1093/jac/dkag308 (PMC13531238; doi:10.1093/jac/dkag308)
Supplement: Supplementary file 1 [file dkag308_supplementary_data.docx]

Table S1 Antimicrobial susceptibility of isolates not susceptible to ceftazidime-avibactam and meropenem-vaborbactam stratified by species.

| **Antimicrobial agent** | **MIC in mg/L** | | |  | **Susceptibility per CLSI^a^** | | |  |
| --- | --- | --- | --- | --- | --- | --- | --- | --- |
|  | **MIC_50_** | **MIC_90_** |  |  | **%S** | **%I** | **%R** |  |
| *K. pneumoniae* (49) |  |  |  |  |  |  |  |  |
| Aztreonam-avibactam | 0.25 | 0.5 |  |  | 98.0 | 2.0 | 0.0 |  |
| Ceftazidime-avibactam | >32 | >32 |  |  | 0.0 |  | 100.0 |  |
| Meropenem-vaborbactam | >32 | >32 |  |  | 0.0 | 8.2 | 91.8 |  |
| Imipenem-relebactam | >8 | >8 |  |  | 0.0 ^d^ | 2.0 | 98.0 |  |
| Cefiderocol | 2 | 16 |  |  | 87.8 | 2.0 | 10.2 |  |
| Levofloxacin | 32 | >32 |  |  | 12.2 | 18.4 | 69.4 |  |
| Gentamicin | 1 | >16 |  |  | 55.1 | 0.0 | 44.9 |  |
| Amikacin | 16 | >32 |  |  | 42.9 | 4.1 | 53.1 |  |
| *E. coli* (24) |  |  |  |  |  |  |  |  |
| Aztreonam-avibactam | 1 | 8 |  |  | 87.5 | 12.5 | 0.0 |  |
| Ceftazidime-avibactam | >32 | >32 |  |  | 0.0 |  | 100.0 |  |
| Meropenem-vaborbactam | >32 | >32 |  |  | 0.0 | 8.3 | 91.7 |  |
| Imipenem-relebactam | 8 | >16 |  |  | 4.2 ^d^ | 0.0 | 95.8 |  |
| Cefiderocol | 2 | 4 |  |  | 91.7 | 4.2 | 4.2 |  |
| Levofloxacin | 16 | 32 |  |  | 4.2 | 0.0 | 95.8 |  |
| Gentamicin | 1 | >16 |  |  | 66.7 | 0.0 | 33.3 |  |
| Amikacin | 4 | 8 |  |  | 58.3 | 33.3 | 8.3 |  |
| *E. cloacae* complex (23) |  |  |  |  |  |  |  |  |
| Aztreonam-avibactam | 0.12 | 0.5 |  |  | 100.0 | 0.0 | 0.0 |  |
| Ceftazidime-avibactam | >32 | >32 |  |  | 0.0 |  | 100.0 |  |
| Meropenem-vaborbactam | 16 | >32 |  |  | 0.0 | 8.7 | 91.3 |  |
| Imipenem-relebactam | 8 | >8 |  |  | 0.0 ^d^ | 0.0 | 100.0 |  |
| Cefiderocol | 4 | 32 |  |  | 52.2 | 21.7 | 26.1 |  |
| Levofloxacin | 1 | 32 |  |  | 39.1 | 13.0 | 47.8 |  |
| Gentamicin | 0.5 | >16 |  |  | 65.2 | 8.7 | 26.1 |  |
| Amikacin | 2 | 8 |  |  | 82.6 | 8.7 | 8.7 |  |
| Other species (19)^c^ |  |  |  |  |  |  |  |  |
| Aztreonam-avibactam | 0.12 | 8 |  |  | 84.2 | 10.5 | 5.3 |  |
| Ceftazidime-avibactam | >32 | >32 |  |  | 0.0 |  | 100.0 |  |
| Meropenem-vaborbactam | 16 | >32 |  |  | 0.0 | 26.3 | 73.7 |  |
| Imipenem-relebactam | >8 | >8 |  |  | 10.5 ^d^ | 0.0 | 89.5 |  |
| Cefiderocol | 1 | 16 |  |  | 84.2 | 5.3 | 10.5 |  |
| Levofloxacin | 2 | 16 |  |  | 42.1 | 5.3 | 52.6 |  |
| Gentamicin | 4 | >16 |  |  | 47.4 | 21.1 | 31.6 |  |
| Amikacin | 4 | 32 |  |  | 68.4 | 21.1 | 10.5 |  |

^a^ Criteria as published by CLSI M100 document (2026)^11^.
^b^ All Enterobacterales species were included in the analysis, but CLSI and EUCAST exclude organisms from the family Morganellaceae.
^c^ Organisms include *Citrobacter freundii* species complex (3), *Klebsiella aerogenes* (3), *K. oxytoca species* complex (5), *Proteus mirabilis* (1), *Providencia rettgeri* (3), *Serratia marcescens* (3), and *Raoultella* spp. (1).
Abbreviations: S, susceptible; I, intermediate; R, resistant.

**Figure S1**. Finlandogram showing the activity of aztreonam-avibactam against selected resistant subsets.

Abbreviations: CAZ-AVI, ceftazidime-avibactam; NS, nonsusceptible; MEM-VAB, meropenem-vaborbactam; CRE, carbapenem-resistant Enterobacterales;
MBL, metallo-β-lactamase.
